# Correlation between the results of in vitro and in vivo chromosomal damage tests in consideration of exposure levels of test chemicals

**DOI:** 10.1186/s41021-018-0094-3

**Published:** 2018-03-06

**Authors:** Eiji Yamamura, Chinami Aruga, Shigeharu Muto, Nobuyuki Baba, Yoshifumi Uno

**Affiliations:** 1 Safety Research Laboratories, Mitsubishi Tanabe Pharma Corporation, 2-2-50 Kawagishi, Toda, Saitama, 335-8505 Japan; 2 Discovery Technology Laboratories, Mitsubishi Tanabe Pharma Corporation, 2-2-50 Kawagishi, Toda, Saitama, 335-8505 Japan

**Keywords:** Genotoxicity, In vitro*-*in vivo correlation, Chromosomal damage, Exposure level

## Abstract

**Introduction:**

We examined the correlation between the results of in vitro and in vivo chromosomal damage tests by using in-house data of 18 pharmaceutical candidates that showed positive results in the in vitro chromosomal aberration or micronucleus test using CHL/IU cells, and quantitatively analyzed them especially in regard to exposure levels of the compounds.

**Findings:**

Eight compounds showed that the exposure levels [maximum plasma concentration (C_max_) and AUC_0-24h_] were comparable with or higher than the in vitro exposure levels [the lowest effective (positive) concentration (LEC) and AUC_vitro_ = LEC (μg/mL) × treatment time (h)]. Among them, 3 compounds were positive in the in vivo rodent micronucleus assays using bone marrow cells. For 2 compounds, cytotoxicity might produce false-positive results in the in vitro tests. One compound showed in vitro positive results only in the condition with S9 mix which indicated sufficient concentration of unidentified active metabolite(s) might not reach the bone marrow to induce micronuclei.

**Conclusion:**

These facts suggested that the in vivo exposure levels being equal to or higher than the in vitro exposure levels might be an important factor to detect in vivo chromosomal damage induced by test chemicals.

## Introduction

For regulatory purposes, genotoxicity data are required to clarify the potential risk of chemicals for the induction of gene mutations and/or chromosomal damages. No single test system is able to detect all genotoxic compounds and therefore, a standard test battery, an in vitro test for gene mutations in bacteria, an in vitro test for chromosomal damage and/or gene mutations in cultured mammalian cells, and an in vivo test for cytogenetic effects in rodent bone marrow cells, is performed usually.

When the standard battery of two or three in vitro genotoxicity tests was performed, it was shown that at least 80% of non-carcinogenic compounds tested gave a false positive result in at least one test [1], and the high rate of “false” or “misleading” positive results from the chromosomal aberration/micronucleus tests would occur when rodent derived cell lines and inappropriate cytotoxicity measures such as relative cell count or replication index are used [2]. In addition, the positive responses in in vitro mammalian cells tests have been the focus of debate because it emerges that assays for genotoxicity in mammalian cells can produce positive responses with chemicals that are not DNA-reactive and do not induce genotoxicity or cancer in vivo, but rather disturb the physiological conditions of the cells in culture, or inflict damage on non-DNA targets and processes within the cell [3].

To further interpret the in vitro positive results, it is suggested that all available data/information of test chemicals should be also considered, such as the pharmacokinetics or pharmacodynamics, the structure–activity relationships, existing knowledge on the mode of action, and the other toxicity testing data*.* [4]. In this study, we focused on in vitro and in vivo exposure levels of test chemicals, because, to the best of our knowledge, extensive analysis from this point of view has not been reported as yet for the relationship between in vitro-in vivo results of chromosomal damage tests. We analyzed it by using our in-house data of pharmaceutical candidates, i.e., quantitative comparison of the lowest effective (positive) concentration of the in vitro chromosomal aberration or micronucleus tests with CHL/IU cells and the plasma concentration of the in vivo rodent chromosomal aberration or micronucleus tests with the bone marrow cells. Furthermore, in order to explore the factors involved in in vitro “irrelevant positive” results, several parameters including indicators of exposure to chemicals in the in vivo and in vitro tests were analyzed.

## Materials and methods

### Test chemicals

Pharmaceutical candidates developed in our company from 2001 to 2017 were reviewed, and 18 compounds were selected for analysis in this study because those had all of data-package required for the analysis, i.e., negative results of bacterial reverse mutation (Ames) test with *Salmonella typhimurium* TA100, TA98, TA1535, TA1537, TA2637, and/or *Escherichia coli* WP2*uvrA*, positive results of in vitro chromosomal aberration (CA) or micronucleus (MN) test using CHL/IU cells, positive or negative results of in vivo (rat or mouse) MN assays with bone marrow cells, and data of toxicokinetics or pharmacokinetics in rodent plasma (Table 1). The compounds consisted of wide range of chemical classes and the modes of action of positive in vitro assays were unknown.

**Table 1:** Results of in vitro and in vivo studies with analyzed 18 compounds

No.	Compound	Ames		in vitro test		in vivo test		Exposure data^a^
1	A	TA100, TA98, TA1537	–	MN/CHL	+	MN/rat	+	Concomitant TK
2	B	TA100, TA98, TA2637, WP2*uvrA*	–	MN/CHL	+	MN/rat	+	PK
3	C	TA100, TA98, TA2637, WP2*uvrA*	–	MN/CHL	+	MN/rat	+	Concomitant TK
4	D	TA100, TA98	–	CA/CHL	+	MN/mouse	–	Concomitant TK
5	E	TA100, TA1535, TA98, TA1537, WP2*uvrA*	–	CA/CHL	+	MN/rat	–	TK
6	F	TA100, TA1535, TA98, TA1537, WP2*uvrA*	–	MN/CHL	+	MN/rat	–	Concomitant TK
7	G	TA100, TA98	–	MN/CHL	+	MN/rat	–	Concomitant TK
8	H	TA100, TA98, TA1537	–	MN/CHL	+	MN/rat	–	Concomitant TK
9	I	TA100, TA1535, TA98, TA1537, WP2*uvrA*	–	CA/CHL	+	MN/rat	–	TK
10	J	TA100, TA1535, TA98, TA1537, WP2*uvrA*	–	CA/CHL	+	CA/rat	–	Concomitant TK
11	K	TA100, TA98	–	CA/CHL	+	MN/rat	–	Concomitant TK
12	L	TA100, TA98	–	CA/CHL	+	MN/rat	–	Concomitant TK
13	M	TA100, TA1535, TA98, TA1537, WP2*uvrA*	–	MN/CHL	+	MN/rat	–	TK
14	N	TA100, TA1535, TA98, TA1537, WP2*uvrA*	–	MN/CHL	+	MN/rat	–	Concomitant TK
15	O	TA100, TA1535, TA98, TA1537, WP2*uvrA*	–	CA/CHL	+	MN/rat	–	Concomitant TK
16	P	TA100, TA1535, TA98, TA1537, WP2*uvrA*	–	MN/CHL	+	MN/rat	–	Concomitant TK
17	Q	TA100, TA1535, TA98, TA1537, WP2*uvrA*		MN/CHL	+	MN/rat	–	Concomitant TK
18	R	TA100, TA98, TA1537	–	MN/CHL	+	MN/rat	–	Concomitant TK

MN, micronucleus test; CA, chromosomal aberration test; CHL, CHL/IU cells; PK, pharmacokinetic study; TK, toxicokinetics study

+, positive; −, negative

^a^In case of no concomitant toxicokinetics study, exposure level was estimated from a reference study by using curve fitting

### Ames test

The methods were essentially same as described previously [5]. In brief, the test chemicals were treated to strains of *S. typhimurium* TA100, TA1535, TA98, TA1537, TA2637 and/or *E. coli* WP2*uvrA* in the presence and absence of a metabolic activation system, a cofactor-supplemented post-mitochondrial fraction prepared from the livers of rats treated with a combination of phenobarbital and β-naphthoflavone (S9 mix) using the pre-incubation method.

### In vitro chromosomal aberration/micronucleus test

The methods were essentially same as described previously [5, 6]. Briefly, the chromosomal aberration test was performed using CHL/IU cells treated with each test chemical for short-term (6 h) in the absence or presence of rat S9 mix followed by 18 h recovery period, or continuously (for 24 h) in the absence of S9 mix and then, were subjected to the microscopic examination for calculation of the incidence of cells with chromosomal aberrations. For the micronucleus test, the cells were treated for short-term (6 h) in the absence or presence of S9 mix followed by 18 or 20 h recovery period, or continuously (for 24 or 26 h) in the absence of S9 mix and thereafter, the incidence of micronucleated cells were analyzed. The highest concentration for the analysis was selected as a concentration showing approximately 50% cytotoxicity that was calculated using relative cell survival (RCC), relative mitotic index (RMI), relative population doubling (RPD) or relative increase in cell count (RICC) in accordance with the previous and the revised ICH-S2 guidelines [7–9], respectively.

### In vivo micronucleus test

Male or female rats (CD/SD or Wister) or mice (CD-1/ICR) were purchased from Charles River Japan Inc. (Tokyo), Charles River Laboratories (Raleigh) or CLEA Japan Inc. (Tokyo), and reared under appropriate housing and feeding conditions. Animal experiments were conducted in accordance with the rules of animal welfare in the testing facilities and approved by the ethical committee. Rats (6–9 weeks old) or mice (7–8 weeks old) were dosed with each test chemical once or repeatedly (two to fourteen daily doses). Preparation of bone marrow samples and the evaluation were performed by the methods as described previously [5] or of Kawabata et al. [10]. In brief, the bone marrow cells were collected at approximately 24 h after the final dosing and were used for the preparation of slide specimens to score the number of micronucleated immature erythrocytes (MNIME). The highest dose for the examination was set as the maximum tolerated dose (MTD) or at 2000 mg/kg/day (the maximum feasible dose, MFD) except for the in vivo positive chemicals (compounds A and C). Compound D decreased the proportion of immature erythrocytes (IME) to total erythrocytes in treated animals at the highest dose slightly, but the change did not inhibit the scoring of MNIME. The others did not reduce the IME ratio at any doses.

### Parameters analyzed

The following data were used for analysis.

In vitro data: Lowest effective (positive) concentration (LEC, μg/mL); Area under the concentration time curve (AUC_vitro_, μg·h/mL) calculated by the following formula on the supposition that the test chemicals were stable in the culture medium, AUC_vitro_ = LEC (μg/mL) × treatment time (h); Relative cell survival%, relative population doubling% or relative increase in cell count% at the LEC (CS% at LEC); Maximum fold increase of MN% or CA%; Treatment condition (short-term with/without S9 mix, continuous without S9 mix).

In vivo data: Maximum plasma concentration (C_max_, μg/mL) and AUC_0-24h_ (AUC_vivo_, μg·h/mL) at the lowest effective dose (LED) in the in vivo positive study or at the highest dose in the in vivo negative study; Time to maximum plasma concentration (t_max_); Plasma protein binding (PPB) %.

## Results

**Fig. 1:**
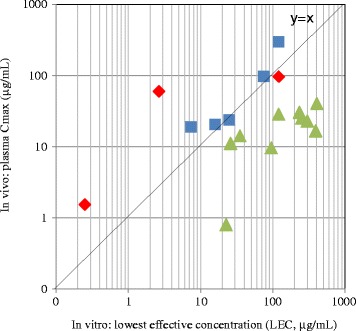
In vitro-in vivo Correlation of Chemical Concentrations in Chromosomal Damage Tests. Red diamond shows in vivo positive compounds with their C_max_ and AUC_vivo_ being equal to or higher than in vitro LEC and AUC_vitro_. Blue square shows in vivo negative compounds with their C_max_ and AUC_vivo_ being equal to or higher than in vitro LEC and AUC_vitro_. Green triangle shows in vivo negative compounds with their C_max_ and/or AUC_vivo_ being less than in vitro LEC and AUC_vitro_

**Fig. 2:**
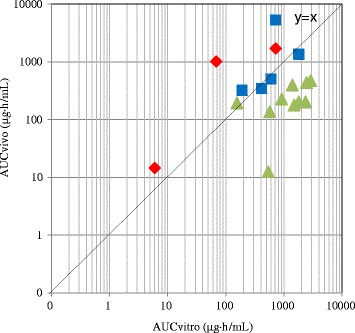
In vitro-in vivo Correlation of Chemical Exposures (AUC) in Chromosomal Damage Tests. Red diamond shows in vivo positive compounds with their C_max_ and AUC_vivo_ being equal to or higher than in vitro LEC and AUC_vitro_. Blue square shows in vivo negative compounds with their C_max_ and AUC_vivo_ being equal to or higher than in vitro LEC and AUC_vitro_. Green triangle shows in vivo negative compounds with their C_max_ and/or AUC_vivo_ being less than in vitro LEC and AUC_vitro_

**Fig. 3:**
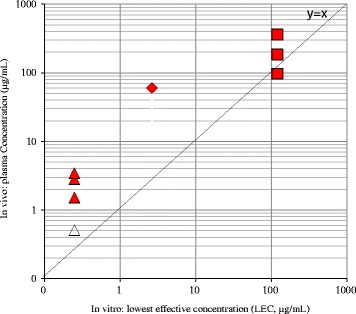
In vitro-in vivo Correlation of Chemical Concentrations in Chromosomal Damage Tests: Dose Response of in vivo Positive Compounds. Diamond indicates compound A. Square indicates compound B. Triangle indicates compound C. Red closed symbols indicate concentrations of in vivo positive doses

**Fig. 4:**
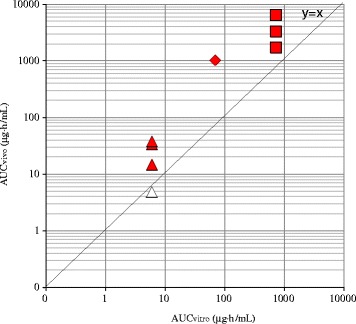
In vitro-in vivo Correlation of Chemical Exposures in Chromosomal Damage Tests: Dose Response of in vivo Positive Compounds. Diamond indicates compound A. Square indicates compound B. Triangle indicates compound C. Red closed symbols indicate concentrations of in vivo positive doses

Eighteen test chemicals showed negative results in the Ames test and positive results in the in vitro CA or MN test (Table 1). In vitro*-*in vivo correlation of chemical exposure levels in the chromosomal damage tests are summarized in Figs. 1 and 2. Based on the in vivo exposure levels, the 18 chemicals were categorized into two types, i.e., type 1: eight chemicals (compounds A, B, C, D, E, F, G and H) of in vivo C_max_ and AUC_vivo_ being equal to or higher than in vitro LEC and AUC_vitro_, respectively, type 2: ten chemicals (compounds I, J, K, L, M, N, O, P, Q and R) of C_max_ and/or AUC_vivo_ being less than in vitro LEC and AUC_vitro_, although the in vivo MN assays were conducted at the MTD or MFD levels (Figs. 1 and 2). The type 1 chemicals included three compounds (compounds A, B, and C) showing positive in the in vivo MN assays (Table 1, Figs. 1 and 2). Regarding the three in vivo positive compounds, the dose-dependency of in vivo exposure levels and the correlation of chemical exposure levels between in vitro and in vivo are summarized in Fig. 3 (LEC vs C_max_) and Fig. 4 (AUC_vitro_ vs AUC_vivo_). As shown, compound C showed in vivo positive responses at the middle and high dose levels but negative at the lowest dose, although the C_max_ and AUC_vivo_ values of all dose levels were equal to or higher than the LEC and the AUC_vitro_.

## Discussions

In this study, eight of the 18 in vitro positive chemicals reached the in vivo exposure levels (C_max_ and AUC_vivo_) being equal to or higher than the in vitro exposure levels (type 1 chemicals), and three of the eight type 1 chemicals showed in vivo positive results in the MN assays. In contrast, no in vivo positive chemical was found in the type 2 chemicals that the in vivo exposure levels (C_max_ and/or AUC_vivo_) were less than the in vitro exposure levels. We have more data-packages of type 2 chemicals (data not shown here because the in vivo MN assays were conducted below the MTD or MFD levels of in vitro positive chemicals), and we could not find any in vivo positive compounds among those types. These facts suggest that the in vivo exposure levels (C_max_ and AUC_vivo_) being equal to or higher than the in vitro exposure levels might be an important factor to detect in vivo chromosomal damage induced by test chemicals. This finding also suggests that continuous high exposure of test chemicals should be carefully taken into account when genotoxicity of test chemicals were evaluated with in vivo MN assays. Obviously, more data-packages including Ames test data with 5 strains would be required to clarify the in vitro*-*in vivo correlation discussed here. However, in our experiences of genotoxicity screening for pharmaceutical candidates, in vivo positives are rarely noted although in vitro positives are often observed, and thus it is difficult to collect and analyze additional cases. Therefore, we hope that similar analysis will be performed in the other test facilities, especially in pharmaceutical companies, because toxico- or pharmaco-kinetic data are usually available only in pharmaceutical candidates.

Regarding five type 1 compounds (D, E, F, G and H) being in vivo negative, their exposure parameters and in vitro test parameters were compared with the in vivo positive cases (Tables 2 and 3). Two chemicals (compounds D and G) were only positive with less than 60% of cell survival using the cytotoxicity index of RCC in the in vitro tests. We retrospectively estimated the RICC for compounds evaluated using RCC data by the method of Fujita et al. [11] (Tables 2, 3 and 4). The positive concentrations of compounds D and G showed less than 50% of RICC (Table 3). These facts indicated the cytotoxicity might produce false-positive results for the 2 chemicals, while the other compounds yielded positive response at concentrations of moderate cytotoxicity. Compound E showed in vitro positive results only under the condition with S9 mix and thus, sufficient concentration of unidentified active metabolite(s) might not reach the bone marrow to induce micronuclei. There were no obvious differences in the other parameters, i.e., t_max_, PPB% and fold increase of MN% or CA% in comparing with those of in vivo positive compounds (Tables 2 and 3). Based on the above discussion and the knowledge from the three in vivo positive chemicals, compounds F and H (and D and G showing in vitro positive response at severe cytotoxic concentration) may be considered as clearly in vivo negative compounds.

**Table 2:** Comparison of parameters between in vivo positive and in vivo negative compounds - in vivo positive case with C_max_ and AUC_vivo_ being equal to or higher than in vitro LEC and AUC_vitro_ -

Compound	LEC (μg/mL)	C_max_ (μg/mL)	AUC_vitro_ (μg·h/mL)	AUC_vivo_ (μg·h/mL)	the highest dose in vivo	t_max_ (h)	PPB (%)	in vitro positive condition	CS at LEC^a^ (%)	Fold increase^b^
A	2.65	59.9	69	1020	–	7.0	98.9	short (+/− S9) continuous^c^	RCC: 87 (RICC: 83)^d^	16.8
B	121	96.5	726	1719	MFD	24.0	86.2	short (− S9)	RCC: 84 (RICC: 79)^d^	3.89
C	0.250	1.53	6	14.6	–	3.0	98.6	continuous	RCC: 92 (RICC: 89)^d^	8.0

LEC, the lowest effective (positive) concentration in the in vitro test; C_max_, maximum plasma concentration; AUC, area under the concentration time curve; MFD, the maximum feasible dose (2000 mg/mg); t_max_, time to maximum plasma concentration; PPB, the ratio of plasma protein binding; short (+/-S9), short-term with/without S9 mix; continuous, continuous without S9 mix

^a^Cell survival ratio at LEC compared to concurrent vehicle control; RCC, relative cell survival; RICC, relative increase in cell count

^b^Maximum fold increases of the incidence of micronucleated cells or cells with chromosomal aberration compared to the concurrent vehicle control value

^c^The data of marked treatment condition showing positive responses with the lowest exposure levels were adopted for the comparison

^d^Estimated RICC from RCC data

**Table 3:** Comparison of parameters between in vivo positive and in vivo negative compounds - in vivo negative case with C_max_ and AUC_vivo_ being equal to or higher than in vitro LEC and AUC_vitro_ -

Compound	LEC (μg/mL)	C_max_ (μg/mL)	AUC_vitro_ (μg·h/mL)	AUC_vivo_ (μg·h/mL)	the highest dose in vivo	t_max_ (h)	PPB (%)	in vitro positive condition	CS at LEC^a^ (%)	Fold Increase^b^
D	75.0	96.8	1800	1360	MFD	5.0	93.2	continuous	RCC: 59 (RICC: 39)	33.0
E	120	298	720	5351	MTD	8.0	99.8	short (+ S9)	RCC: 74 (RICC: 61)	ND
F	7.34	19.0	191	323	MTD	24.0	98.6	continuous	RCC: 81 (RICC: 74)	6.12
G	24.9	23.6	598	508	MTD	4.0	98.5	continuous	RCC: 48 (RICC: 31)	3.0
H	15.9	20.5	413	346	MTD	7.0	91.5	continuous	RICC: 71	3.0

LEC, the lowest effective (positive) concentration in the in vitro test; C_max_, maximum plasma concentration; AUC, area under the concentration time curve; MFD, the maximum feasible dose (2000 mg/mg); MTD, the maximum tolerated dose; t_max_, time to maximum plasma concentration; PPB, the ratio of plasma protein binding; CS, the ratio of cell survival; short (+S9), short-term with S9 mix; continuous, continuous without S9 mix; ND, not determined due to the vehicle control value “0”

^a^Cell survival ratio at LEC compared to concurrent vehicle control. RCC, relative cell survival; RICC, relative increase in cell count

^b^Maximum fold increases of the incidence of micronucleated cells or cells with chromosomal aberration compared to the concurrent vehicle control value

Estimated RICC from RCC data

**Table 4:** Comparison of parameters between in vivo positive and in vivo negative compounds - in vivo negative case with C_max_ and/or AUC_vivo_ being less than in vitro LEC and AUC_vitro_ -

Compound	LEC (μg/mL)	C_max_ (μg/mL)	AUC_vitro_ (μg·h/mL)	AUC_vivo_ (μg·h/mL)	the highest dose in vivo	t_max_ (h)	PPB (%)	in vitro positive condition	CS at LEC^a^ (%)	Fold Increase^b^
I	250	25.1	1500	181	MTD	0.3	90.9	short (+*/−* S9)	RCC: 83 (RICC: 75)**	9.7
J	22.5	0.8	540	12.6	MFD	8.0	90.1	continuous	RMI: 70	ND
K	300	22.9	1800	205	MTD	2.0	92.1	short (− S9)	RCC: 42 (RICC: 14)**	ND
L	95	9.6	570	138	MFD	8.0	96.3	short (+*/− S9)	RCC: 55 (RICC: 33)**	ND
M	392	16.7	2350	204	MTD	–	92.8	short (+*/− S9)	RCC: 57 (RICC: 43)**	24.7
N	26	11.1	156	196	MFD	7.0	98.7	short (− S9)	RCC: 46 (RICC: 28)**	18
O	120	28.5	2880	475	MTD	4.3	83.0	short (+/− S9) continuous*	RPD: 57	ND
P	35.1	14.2	913	229	MFD	3.0	67.2	continuous	RICC: 67	3.4
Q	407	40.5	2442	441	MFD	3.0	89.6	short (+ S9)	RICC: 70	3.1
R	233	30.3	1398	400	MTD	1.0	93.9	short (+*/− S9)	RICC: 65	6

LEC, the lowest effective (positive) concentration in the in vitro test; C_max_, maximum plasma concentration; AUC, area under the concentration time curve; MFD, the maximum feasible dose (2000 mg/mg); MTD, the maximum tolerated dose; t_max_, time to maximum plasma concentration; PPB, the ratio of plasma protein binding; CS, the ratio of cell survival; short (+/− S9), short-term with/without S9 mix; continuous, continuous without S9 mix; ND, not determined due to the vehicle control value “0”

^a^Cell survival ratio at LEC compared to concurrent vehicle control. RCC, relative cell survival; RMI, relative mitotic index; RICC, relative increase in cell count; RPD, relative population doubling

^b^Maximum fold increases of the incidence of micronucleated cells or cells with chromosomal aberration compared to the concurrent vehicle control value

^*^The data of marked treatment condition showing positive responses with the lowest exposure levels were adopted for the comparison. The lowest exposure levels were same for compound J in the short-term treatment conditions with and without S9 mix

**Estimated RICC from RCC data

Generally, in vitro assays are used as the initial genotoxicity screening. When a positive result is obtained, we consider the mode of action (MOA) to evaluate the nature of the genetic activity and select follow-up tests. If a MOA is unclear, or a hypothesized MOA is not supported by sufficient data, a weight of evidence (WOE) approach can be followed to guide the interpretation of the results by performing in vivo tests with a same endpoint reflecting the damage found in the initial screening assay, that is, an in vitro positive result of a test chemical is a trigger to conduct the 2nd in vivo genotoxicity assay [9]. When two in vivo negative data are obtained under the suitable test conditions (e.g., use of MTD levels), the in vitro positive result would be considered as non-relevant to in vivo. However, based on the knowledge noticed in this study, the non-relevancy may be questionable in cases that the in vivo exposure levels are less than the in vitro exposure levels. On the other hand, when it is practically impossible to use a dose-level that the in vivo exposure level is equal to or more than the in vitro exposure levels, e.g. such a dose-level is above the MTD, judgement of non-relevant to in vivo seems still acceptable because there is no alternative approach to assess in vivo genotoxicity. From another point of view, when an in vivo negative result is obtained at sufficient exposure levels above the in vitro positive ones like compounds F and H in this study, one negative result in one in vivo assay might be a sufficient evidence to conclude non-relevant to in vivo. To clarify this consideration, we need, at least, to show negative results of compounds F and H with a 2nd in vivo genotoxicity test (e.g., in vivo comet assay with liver and stomach). More case-studies would be clearly needed to justify these considerations.

## References

[1] Kirkland D, Pfuhler S, Tweats D, Aardema M, Corvi R, Darroudi F (2007). How to reduce false positive results when undertaking *in vitro* genotoxicity testing and thus avoid unnecessary follow-up animal tests: report of an ECVAM workshop. Mutat Res.

[2] Fowler P, Smith R, Smith K, Young J, Jeffrey L, Carmichael P (2014). Reduction of misleading (“false”) positive results in mammalian cellgenotoxicity assays. III: sensitivity of human cell types to known genotoxic agents. Mutat Res.

[3] Fowler P, Smith R, Smith K, Young J, Jeffrey L, Kirkland D (2012). Reduction of misleading (“false”) positive results in mammalian cell genotoxicity assays. II. Importance of accurate toxicity measurement. Mutat Res.

[4] Dearfield KL, Thybaud V, Cimino MC, Custer L, Czich A, Harvey JS (2011). Follow-up actions from positive results of *in vitro* genetic toxicity testing. Environ Mol Mutagen.

[5] Yamamura E, Muto S, Yamada K, Sato Y, Iwase Y, Uno Y (2015). Chromosomal damage and micronucleus induction by MP-124, a novel poly(ADP-ribose) polymerase-1 (PARP-1) inhibitor: evidence for a non-DNA-reactive mode of action. Mutat Res.

[6] Kato K, Yamamura E, Kawanishi M, Yagi T, Matsuda T, Sugiyama A (2011). Application of the DNA adductome approach to assess the DNA-damaging capability of *in vitro* micronucleus test-positive compounds. Mutat Res.

[7] International Conference on Harmonization (ICH) of Technical Requirements for Registration of Pharmaceuticals for Human Use. Genotoxicity: Guidanceon Specific Aspects of Regulatory Genotoxicity Tests for Pharmaceuticals. S2A (1995).

[8] International Conference on Harmonization (ICH) of Technical Requirements for Registration of Pharmaceuticals for Human Use. Genotoxicity: A Standard Battery for Genotoxicity Testing of Pharmaceuticals. S2B (1997).

[9] International Conference on Harmonization of Technical Requirements for Registration of Pharmaceuticals for Human Use ICH Harmonised Tripartite Guideline, Guidance on Genotoxicity Testing and Data Interpretation for Pharmaceuticals Intended for Human Use S2(R1), 2011.

[10] Kawabata M, Takasawa H, Minowa S, Saitou H, Nakagawa M, Nakajima M (2007). New method of specimen preparation as cell suspension for a micronucleus test. Genes and. Environment.

[11] Fujita Y, Kasamatsu T, Ikeda N, Nishiyama N, Honda HA (2016). Retrospective evaluation method for in vitro mammalian genotoxicity tests using cytotoxicity index transformation formulae. Mutat Res.

